# Sedo-analgesia in double lumen enteroscopy

**DOI:** 10.4103/0972-9941.33279

**Published:** 2007

**Authors:** Dinesh K Singh, Arpan Chakraborty, Rahul Dutta

**Affiliations:** Department of Anesthesiology, Institute of Medical Sciences, Banaras Hindu University, Varanasi, Uttar Pradesh, India

Double lumen enteroscopy first described by Yamamoto and colleagues in 2001,[1] is an exciting new endoscopic technique that allow complete visualization of the small intestine by using a 200 cm enteroscope equipped with a 140 cm over-tube used either by anterograde or retrograde technique according to location of the lesion with a mean examination time of 60-90 min. Monitored anesthesia care during this procedure is of paramount importance for successful completion as significant subset of patients is unable to tolerate the procedure without sedation. Sedatives and hypnotics are used to make the procedures more tolerable for patients by reducing anxiety and providing a degree of intraoperative amnesia while allowing them to rest during the operation.[2]

The patients (n=52) were prepared with overnight fasting, intravenous fluid started and ondansetron 0.1 mg/kg i.v, given before the procedure. Left lateral position was given to facilitate drainage of secretion with knees bent toward the chest and head flexed in forward position with a pillow placed behind the patient back. A bolus dose of 1% propofol 0.5 mg/kg[3] and fentanyl 1 mcg/kg was given followed by an infusion of propofol at a rate of 25 mcg/kg/min till the end of procedure, keeping the level of sedation between Ramsay sedation score 2 to 3. Supplemental oxygen can be administered through nasal cannula at 1- 2 L/min. Continuous pulse oximetry, heart rate and noninvasive blood pressure at three min interval were monitored throughout the procedure. This procedure can be performed by two methods, anterograde and retrograde approach. Time duration of the two methods is almost same in experienced hands, 45 min to one hour. Total dose of propofol required was between100 to 150 mg in adult patients. Position of the patient with retrograde approach was changed from lateral to lithotomy for facilitation of the further insertion of enteroscope during the procedure. There may be requirement of anticholinergics to relieve smooth muscle spasm to facilitate the passage of enteroscope. Following the completion of the procedure the patient was transferred to recovery room and observed for oxygen saturation, blood pressure, pulse rate and pain score (Visual Analog Scale) and nausea and vomiting. No significant hemodynamic variation was noted during the procedure. Absence of nausea and vomiting, visual analog scale less than 5, ability to void and a conscious and cooperative mental state were used as discharge criteria.

Sporea *et al.*[4] mentioned in their study that sedo-analgesia during colonoscopy seems to be better in order to ensure comfort to the patient and a high quality examination than sedation alone In our cases, the combination of propofol, a hypnotic drug, appropriate for short lasting anesthesia and fentanyl with intense analgesic effect and brief duration of action, enabled the gastroenterologist to perform the procedure easily without any discomfort to the patient and can increase the tolerance of patient for subsequent endoscopic examination, if needed. Propofol, for gastro-intestinal endoscopy, administered by registered nurses under the supervision of endoscopists has proven safe, but is not realistically feasible in most US endoscopy units. Therefore, propofol in the United States is being administered almost entirely by anesthetists as in our study.[5]
